# Complaints about Violations of Voluntary and Pharmaceutical Industry-Run Medicine Promotion Codes in Canada

**DOI:** 10.1177/27551938231165158

**Published:** 2023-03-20

**Authors:** Joel Lexchin

**Affiliations:** 1 School of Health Policy and Management, 7991 York University, Toronto, Canada; 2 Faculty of Medicine, University of Toronto, Toronto, Canada

**Keywords:** Canada, promotion codes, pharmaceutical industry, complaints, penalties

## Abstract

Although regulation of pharmaceutical promotion in Canada is covered under the Food & Drugs Act and Regulations, in practice it is regulated by two codes, one a code administered by the Pharmaceutical Advertising Advisory Board and a second one controlled by Innovative Medicines Canada, the lobby organization for the large majority of the Canadian and foreign-owned pharmaceutical companies operating in Canada. This study examines complaints about code violations between 2012 and 2021 and puts those complaints and their outcomes into the context of the stringency of the codes, their governance, monitoring, penalties for violations, and compliance by companies. It combines the findings from this analysis with international experience with industry-run codes and concludes that overall, the Canadian codes are ineffective in controlling promotion. Finally, it offers recommendations for how regulation can be improved and how doctors’ reliance on information from pharmaceutical companies can be reduced.

In Canada, pharmaceutical manufacturers are prohibited under the Food and Drugs Act (F&DA) and Regulations from presenting their products in a misleading light by underplaying the negative effects and overemphasizing the positive.^1,2^ In the F&DA, advertising is defined as “any representation by any means whatever for the purpose of promoting directly or indirectly the sale or disposal of any food, drug, cosmetic or device.” Despite having the nominal control over promotion, over the past century, Health Canada has done little to exercise that power. Health Canada's position is that “it's not our policy to treat advertising as the definitive source of information with respect to drugs”.^3^ The agency explicitly practices a form of “delegated” self-regulation, a regulatory model also seen in Australia^4^ and most European countries,^5^ including the United Kingdom.^6^ According to Health Canada, “[w]hile it is the responsibility of Health Canada to administer the Food and Drugs Act and related Regulations, it is the responsibility of market authorization holders (manufacturers and distributors) to ensure that their advertising complies with the relevant legislation and regulations”.^7^

In practice, having market authorization holders regulate their promotion has meant that Health Canada has voluntarily agreed to turn over the day-to-day regulation of promotion of prescription drugs to a combination of an industry association (Innovative Medicines Canada [IMC]) that practices self-regulation of promotion and an independent, multistakeholder group (Pharmaceutical Advertising Advisory Board) with strong representation from organizations that directly profit from promotion. In return, industry was willing to take on regulation in order to avoid statutory controls^8^ and to enhance its reputation for ethical behavior, as the following quote illustrates: “the ill-judged and excessive practices of a few…are capable of damaging the image of the entire industry”.^8^

IMC is the organization that represents the large majority of small, Canadian-owned and foreign-owned companies operating in Canada. The primary objective of IMC is to advance the interests of the industry through lobbying around health policy issues and interacting with other stakeholders such as the medical profession and patient groups. IMC, then known as the Canadian Pharmaceutical Manufacturers Association, produced the first version of its Principles of Ethical Drug Promotion in 1959.^9^ The 1966 version of its code was developed “in close co-operation with Health Protection Branch [the branch of government that regulated drugs] officials”.^8^ The code has since been renamed the Code of Ethical Practices and its latest version was released in 2022.^10^ IMC still uses its code to enhance its reputation as can be seen in the use of the term “Ethical Practices” in the title and from the statement about purpose in the current version of the code: “The innovative pharmaceutical industry recognizes that Canadians expect companies to be accountable for their conduct…(the Code) provides a mechanism for Members to establish and maintain an ethical culture through a committed, self-regulated approach”.^11^

In the mid 1970s, Marc Lalonde, then the federal Minister of Health and Welfare, issued an ultimatum to the industry to reform its promotional practices or else face the prospect of government action.^8^ In response, the industry sponsored a sequence of events that resulted in the creation of the Pharmaceutical Advertising Advisory Board (PAAB) in 1975, a multi-stakeholder organization. The PAAB develops and administers its own distinct Code of Advertising Acceptance.^12^ The PAAB is what Health Canada terms an Advertising Preclearance Agency (APA) and operates with the explicit approval of Health Canada, as the following quote from a Health Canada guidance document illustrates. “Canadian APAs provide advertising material review services to advertisers and advertising agencies. They use Health Canada's guidance documents and their own codes of advertising to ensure that advertising material submitted to them, in all media, complies with the F&DA [Food and Drugs Act], CDSA [Controlled Drugs and Substances Act], and respective Regulations”.^13^

Pharmaceutical companies in Canada spend close to $500 million annually to promote their products to Canadian physicians.^14^ The companies often claim that the purpose of their promotion is to bring their drugs to the attention of doctors and to inform doctors about them,^15^ as the following quote from IMC demonstrates: “Provider-supported detailing generates awareness about new treatments and provides science-based and Health Canada approved advice on how to administer these medications”.^16^ However, a systematic review of the international literature failed to find any beneficial effects on prescribing from interactions between doctors and pharmaceutical companies.^17^

The controversy about whether drug promotion benefits or harms prescribing hinges, in part, on the accuracy of information in promotion about indications for use and the outcomes of drug treatments. In order to ensure accuracy, it is important that the information in promotion is complete and objective. An evaluation of the functioning of the two Canadian codes in 1997 found substantial weaknesses in their enforcement,^18^ and subsequent research in other countries has also documented deficiencies in industry codes.^19^ The purpose of this study is two-fold: to report on the characteristics of the Canadian codes as they relate to their effectiveness in regulating promotion and to assess how often the codes were reported to be violated and what penalties are imposed for those violations.

## Methods

Information about the codes, including who is responsible for their governance, whether compliance is voluntary or compulsory, the types of promotion covered, how revisions are enacted and compliance is monitored, who adjudicates complaints, and what penalties are levied, was gathered from the information in the codes on the IMC and PAAB websites.^10,12^

Information about violations and penalties between 2012 to 2021 came from annual reports from the two organizations.^10,20^ PAAB reports for the years 2012–2018, except for 2015, were in quarterly newsletters. There were annual reports for 2015 and 2019­­­–2021. The annual number of individual submissions to the PAAB was sourced from the PAAB website.^21^

Information was gathered between October 11–14, 2022, by a single individual. No ethics approval was required as all data were publicly available. Only descriptive data are reported. All dollar amounts are in Canadian dollars.

## Results

Table 1 gives details about the characteristics of the two codes. Both codes are governed by the boards of the respective organizations. Although the PAAB cannot enforce compliance with its code, IMC has made compliance with both codes compulsory for its membership, although compliance with both codes is voluntary for non-members. The IMC code “applies to the activities of all Member employees who interact with Stakeholders for the purpose of commercializing prescription medicines” such as activities of sales representatives, sponsorship of continuing medical education, displays at medical conferences, and interactions with patient groups.^11^ The PAAB code “applies to all communications in which claims, quotes and references are made for health care products or in relation to health care product issues”.^12^ The process for revising the IMC code was not stated, while the PAAB solicits submissions from outside stakeholders. Revisions to the PAAB code require a two-thirds majority of the board. Compliance with both codes is determined through complaints and the PAAB also monitors journals and the internet for compliance, although no further details were given about how the monitoring takes place. There is no information about who is eligible to file a complaint to IMC. Complaints to the PAAB can be filed by health professionals; health care organizations; pharmaceutical companies; federal regulatory bodies, including Health Canada; and drug payer organizations, including provincial ministries of health.

**Table 1.** Characteristics of the Codes from Innovative Medicines Canada (IMC) and the Pharmaceutical Advertising Advisory Board (PAAB).

Code	Governance	Obligation to comply	Areas covered	Process for revisions	Monitoring of compliance	Adjudication	Penalties
IMC	Board of Directors (representatives from member companies)	IMC members required to comply, voluntary for other companies	Activities of all member employees who interact with stakeholders for the purpose of commercializing prescription medicines, excluding medical devices and over-the-counter products, through interactions between members and stakeholders	Not stated	Complaints; no information about who is eligible to file a complaint	No dialogue between complainant and company before the complaint is considered. Industry Practices Review Committee^a^ [IPRC] reviews complaint; appeal to committee composed of a representative of the IPRC and each of parties involved in the complaint plus three adjudicators	Range: $25 000 for first violation to $100 000 for fourth violation within a year, plus posting on IMC website for up to 24 months; if five or more violations within a year 12-month probationary period of IMC membership; expulsion from IMC if further violation within the probationary period
PAAB	13-member board^b^	IMC members required to comply, voluntary for other companies	Any paid message communicated by Canadian media, with the intent to influence the choice, opinion or behavior of those addressed by commercial messages	Submissions from multiple stakeholders; revisions require a two-thirds majority of the board	Preclearance; complaints; regular monitoring of journal ads and the internet plus receipt of direct-mail/detail aid materials collected by health professionals; complaints can be filed by health professionals, health care organizations, pharmaceutical companies, federal regulatory bodies including Health Canada and drug payer organizations including provincial ministries of health	Initial dialogue between complainant and company required before Commissioner considers complaint; appeal to Review Panel composed of three PAAB Board members; if advertiser does not comply with ruling then escalation to Health Canada	Range: immediate withdrawal of offending advertising; notices in annual reports or newsletters; public letters of apology

^a^ Permanent members: two Member representatives and two external representatives, health care professionals appointed by the Board of Directors, a representative appointed by IMC's President and IMC's general counsel; additional members: one individual appointed by the IMC President, one representative from the Pharmaceutical Advertising Advisory Board (PAAB), one external representative from the scientific community appointed by the Industry Practices Review Committee (IPRC).

^b^ Representatives from: Canadian Association of Medical Publishers, Canadian Medical Association, Food Health & Consumer Products of Canada, BIOTECanada, Canadian Dental Association, Best Medicines Coalition, Canadian Healthcare Communications Providers, Canadian Pharmacists Association, Consumer Council of Canada, Canadian Generic Pharmaceutical Association, Innovative Medicines Canada.

All communications need to be prescreened by the PAAB staff before companies can use them, and the membership of the Canadian Association of Medical Publishers has agreed not to publish ads that have not first been prescreened. The Industry Practices Review Committee, the majority of whose members come from IMC, adjudicates complaints about violations of the IMC code, and decisions can be appealed. There is no initial dialogue between complainants and the company before the IMC considers a complaint, but the PAAB does require dialogue between the two parties. The PAAB Commissioner adjudicates complaints about its code, with appeals allowed, and if the advertiser does not comply with the ruling, complaints can be referred to Health Canada, something that does not seem to happen when there are disputes about IMC rulings. Finally, IMC can level monetary penalties of up to $100 000. It also reports violations on its website and can suspend a company's membership in the organization. In contrast, the PAAB relies on non-monetary penalties.

There were a total of six complaints about violations of the IMC code in 2012, 2015, 2016, 2017, and 2018 but none were upheld (Table 2). One complaint was made by an individual and the other five were made by a competitor company. In three years (2013, 2014, and 2017), there were no complaints and from 2019 onward there was no reported information about complaints. No decisions were appealed. Because no complaints were upheld, no company names were published. Furthermore, no complaints were identified as coming from internal company sources. (Supplementary Table 1 gives all information extracted about the complaints.)

**Table 2.** Complaints and Violations of the Innovative Medicines Canada Code, 2012–2021.

Year	Number of complaints	Number of complaints upheld	Penalty	Number of appeals
2012	1	0		0
2013	0			
2014	0			
2015	1	0		0
2016	3	0		0
2017	0			
2018	1	0		0
2019	No information reported			
2020	No information reported			
2021	No information reported			

PAAB only reports about “Stage 2” complaints—that is, where initial dialogue between the complainant and the company being accused of violating the code does not resolve the issue. One complaint could contain multiple allegations about a single piece of communication. Table 3 summarizes information about complaints to the PAAB. There were 42 complaints involving 114 separate allegations (range 1–9 allegations per complaint), but only 12 (29.3%) of the complaints involved ads that had been prescreened. (One complaint could not be analyzed as there was no information about it.) All the names of the companies were published regardless of whether the complaints were upheld. Sixteen of the 41 companies were IMC members. Significantly less than 1 percent of all submissions to the PAAB for prescreening eventually resulted in complaints. In 2019, there were nine complaints filed, the most in a single year. None of the 41 complaints were reported as coming from PAAB monitoring of journal ads or the internet. Six complaints came from health care professionals; the rest came from competitor companies, but as with complaints to IMC, no complaints to the PAAB were from internal company sources. Eighty-one of the 114 (71.1%) allegations were upheld. Although the PAAB can require companies to issue a public letter of apology, this particular penalty was not used. The PAAB did require the immediate withdrawal of the material involved in 17 allegations, but because the material was no longer being used, no penalty was levied in four of the 41 cases. Only one ruling was appealed. The PAAB sent four complaints to Health Canada, but there was no information in any PAAB publication about what action Health Canada took. (Supplementary Table 2 gives all information extracted about the complaints.)

**Table 3.** Complaints and Violations of the Pharmaceutical Advertising Advisory Board Code, 2012–2021.

Year	Number of submissions to PAAB	Number of complaints, n (percent of submissions)	Number of communications with complaints that were precleared by PAAB	Allegations		Range of Penalties
				Total number	Number upheld	
2012	6691	4 (0.06)	2	5	3; 2 sent to Health Canada	Cease distribution, withdraw ad
2013	7180	2 (0.03)	0	2	1; 1 sent to Health Canada	Website shut down
2014	7050	2^a^ (0.03)	1	3	0	
2015	7718	6 (0.08)	2	7	6	Company required to add additional footnotes, cease making claims and recall material, cease distributing material, not documented
2016	7535	3 (0.04)	0	6	6	Provide PAAB with evidence of withdrawal of material, provide PAAB with proof of actions taken, Health Canada informed, if PAAB reviewed material would ask for “cover to cover” revision
2017	7412	5 (0.07)	0	13	11; 2 rulings unclear	Correct website, refer to Health Canada, submit leaflet for review, withdraw APS^b^ from the marketplace; one ruling appealed
2018	7965	3 (0.04)	1	6	2	PAAB will not renew APS when they expire, stop distribution and collect and destroy any material company still has
2019	7635	9 (0.12)	3	52	36	Cease use of APS, APS no longer used and no immediate action needed, referred to Health Canada, immediately take down relevant portion of website
2020	9080	2 (0.02)	1	4	4	Future changes will be made to PAAB code, material no longer being used and therefore penalty unnecessary
2021	10546	5 (0.05)	2	16	8; 5 partially upheld	No immediate action needed, material not to be used in future, remove ad immediately,

^a^ No information about one complaint.

^b^ Advertising/promotion systems.

## Discussion

Over the 10-year period covered by this study, there were a total of 47 complaints about code violations: six of the IMC code and 41 of the PAAB code. None of the complaints about violations of the IMC code were upheld. The 41 complaints about violations of the PAAB code included 114 allegations, of which 71.1 percent were upheld, and only a very small fraction of the annual submissions that the PAAB received resulted in complaints. However, an unknown number of complaints about PAAB code violations could have been resolved through dialogue and would therefore not have been reported.

Because there were no complaints upheld by the IMC Industry Practices Review Committee, the type of penalty imposed could not be evaluated. The finding that 40 of all 47 complaints originated from competitors is congruent with the concept that the aim of self-regulation or regulation with a substantial industry input (PAAB) is actually the control of anti-competitive practices–that is, maintaining a level playing field for companies, rather than improving the ethical behavior of companies.

The annual number of reported complaints and breaches of the two Canadian codes are in line with those about the code maintained by Medicines Australia, the industry body in that country. In 2018–2019, there were three complaints about all forms of promotion and one was upheld. In 2019–2020, there were four complaints and all were upheld.^22^ In contrast, in the United Kingdom in 2018, 2019, and 2020, there were 87, 132, and 148 complaints received and 60 (69.0%), 96 (72.7%), and 86 (58.1%) were upheld, respectively.^23^

The findings of this study show that the predominant features characterizing the IMC and PAAB codes are minimal to non-existent outside involvement in their governance, development, and revision; the use of complaints to detect violations; and the structuring of penalties so as not to impose a significant burden on companies that violate the provisions of the codes.

Industry-run codes in other industries typically are weak and allow participant companies to maintain practices that are widely seen as hard to justify.^24^ In the 1980s, the Canadian Tobacco Manufacturers’ Council voluntary code banned tobacco ads on billboards within 200 meters of schools. When Monique Begin, the Health Minister at the time, complained that this ban was not being observed, the tobacco industry blamed advertising agencies and billboard companies.^25^

Products made by the pharmaceutical industry are not comparable to those made by the tobacco industry, but the limitations of voluntary industry codes are similar, for example, in the way that industry tries to deny the applicability of codes. In 2018, Health Canada wrote to opioid manufacturers and distributors asking them to immediately stop promoting the drugs to health care workers while the government considered introducing new regulations.^26^ In its response, available through a previously released Access to Information request, IMC maintained that reimbursement for travel and hospitality expenses to attend industry-sponsored events, as well as gifts of meals and equipment, could not be considered advertising.^27^

The number of complaints and violations do not, a priori, necessarily reflect the effectiveness of codes in ensuring accurate and complete information in promotion. The number is related to four factors: (*a*) the stringency of the code, (*b*) how the code is administered, (*c*) how vigorously the code is monitored and the severity of penalties imposed, and (*d*) how compliant companies are with the provisions of the code. The interpretation of the number of complaints and violations of the IMC and PAAB codes need to be seen in light of these four factors.

### Stringency of Voluntary and Industry-Run Codes

There are two major theoretical reasons to favor industry codes over government regulation. First, self-regulation is less expensive for government as regulatory agencies rarely have the resources to make it economically rational for individual firms not to cheat. Second, industry has more expertise and up-to-date knowledge about industry practices than government so as to enable it to provide effective regulation.^28^ Both the British Association of the British Pharmaceutical Industry (ABPI) and the separate body that administers its code, the Prescription Medicines Code of Practice Authority, have repeatedly stated that the “integrity of self-regulation” is underpinned by an assumption that companies can be relied on to provide “complete and accurate information”.^29^

Voluntary self-regulation, therefore, seems an attractive option because, lacking government–industry adversariness, it could be a more flexible and cost-effective option. However, although industry self-regulation may temper some of the excesses in advertising, it may also allow practices that are beneficial for business across the industry. For example, if it allows the indications for use for a statin to be presented more broadly than is reflected in approved product information, all statin manufacturers may benefit. Similarly, if the industry code allows advertising that downplays serious harmful effects of medicines by only including these risks in fine-print product information, all manufacturers potentially benefit from the exaggerated view of benefit as compared with harm. From the business perspective, self-regulation is mostly concerned with the control of anti-competitive practices. Therefore, when industrial associations draw up their codes of practice, they deliberately make them vague or do not cover certain features of promotion. That allows companies wide latitude for unethical behavior.

Preclearance by the PAAB is a potential strength of its code and may reduce the number of complaints because material, on average, is resubmitted and evaluated twice before it is cleared,^21^ but that has to be balanced against the code's weaknesses. A comparison of the effectiveness of regulation of journal advertising looked at the Australian system of industry self-regulation, the Canadian PAAB, and direct regulation by the United States Food and Drug Administration. Although Canadian ads were marginally more informative than Australian ones, American ads were much better, although they too had their weaknesses.^30^ A comparison of two voluntary codes intended to operate internationally–the World Health Organization's Ethical Criteria for Medicinal Drug Promotion, which is independent of industry influence, and the Code of Marketing Practices from the International Federation of Pharmaceutical Manufacturers and Associations–found that the former covered a wider range of promotional practices and its provisions were more stringent than those of the latter.^31^

There are two significant weaknesses in the IMC code. The first is the frequent use of vague language that can lead to a wide range of interpretations. For example, the code states that “Members may choose to adopt training modules/curricula of third party suppliers/institutions or they may choose to adopt company-generated training materials”; “the [dollar] amount [of meals] should be reasonable”; “Members should take all reasonable measures to ensure that Health Care Professionals are informed in a timely manner with respect to all new product information,” leaving the terms “reasonable” and “timely” open to variable interpretations.

The second major weakness is that the code fails to cover several important areas. There are no provisions about interactions with health care professional students or involvement in their educational activities. There is no provision requiring companies to publicly report each transfer of value to health care professionals, health care institutions, or health care organizations, as occurs in most other industrialized nations.^32^ Additionally, there is no provision about the frequency with which samples can be provided nor the number of samples that can be provided, beyond the meaningless phrase that it should be a “limited quantity.”

A major weakness in the PAAB code is that while it requires key risk information to be included in ads, any information that does not appear in the Health Canada-approved product information (Product Monograph [PM], called the “label” in the United States and the Summary of Product Characteristics in Europe) does not have to appear in ads. Therefore, if the PM lacks a statement saying that the medication does not reduce morbidity and/or mortality, then advertisements do not have to contain a statement to this effect.

While the PAAB code requires a legible font size for print ads, there is no requirement to make the generic name of the drug the same size as the brand name. Additionally, the generic name does not have to be used each time that the brand name is given despite evidence that use of the generic name leads to better prescribing.^17^ Companies are allowed to make statements in journal ads about the effects of drugs, even if the clinical significance of those effects is unknown, as long as the ad also includes that caveat. The code does require a “fair balance of risk to benefit,” but there is no specific requirement that equal space in the ads be devoted to harms and benefits and there is no provision requiring the font size used to describe benefits and harms to be equal.

### Administration

The administration of the IMC code is a closed process, in the sense that there does not seem to be any outside consultation in its revision. The majority of the members of its Industry Practices Review Committee, which adjudicates complaints, comes from IMC, and the membership of the Board of Directors that governs the code comes from IMC companies. The lack of any information about the presence or absence of code violations after 2018 raises the question about the transparency of the IMC in providing information about how well its code is operating. If a complaint is not upheld, neither the name of company making the complaint nor the company accused of violating the code are made public.

The PAAB is slightly more open in that it does seek outside input into code revisions and its board includes representatives from a variety of organizations. However, any changes to the PAAB code need to be approved by a two-thirds majority of the 13-member board, which has representatives of five organizations (Canadian Association of Medical Publishers, Food Health & Consumer Products of Canada, BIOTECanada, Canadian Healthcare Communications Providers, and Innovative Medicines Canada) that potentially benefit from advertising. The PAAB is up-to-date in reporting about complaints and violations, but it currently does not provide any details about multiple individual allegations in a single complaint. The Commissioner's remarks are usually of the nature of “X allegations out of Y” were upheld.

### Monitoring of Compliance and Penalties

Vilhelmsson and colleagues, in their evaluation of the code of the ABPI, maintain that “Successful deterrence is based in the first instance on a credible threat of detection, which in turn depends on effective regulatory monitoring and surveillance”^33^ and the strength of penalties for violating a code. The IMC and the PAAB codes rely exclusively or primarily, in the case of PAAB, on complaints for monitoring compliance. The general consensus is that active monitoring is superior to passive (complaints-driven) monitoring in encouraging compliance with regulations.^34,35^

The degree to which scrutiny of journal ads and the internet contributes to enforcement by the PAAB is unknown. The difference between passive and active monitoring in being able to detect code violations is illustrated by the results of a study examining ads for antidepressants in Swedish medical journals. Based on complaints to the two Swedish self-regulatory authorities, there were 23 violations out of 124 unique antidepressant ads. However, when the ads were independently evaluated, an additional 24 violations were detected.^36^

One of the sanctions available to the IMC in cases of code violation is the publication of the violation on the organization's website. Because there were no violations of the IMC code, it is not known if, or how frequently, it makes violations public. According to the Prescription Medicines Code of Practice Authority, the most important sanction available to the U.K. self-regulatory body to discourage violations is adverse publicity.^33^ However, when it comes to violations of the ABPI code's provisions about off-label promotion, 19 of 41 companies in breach over a 10-year period were ruled in breach more than once, and 10 companies were ruled in breach three or more times.^33^ This pattern of repeated violations suggests that adverse publicity may not be effective. In no case where the PAAB upheld a complaint did it require a public letter of apology as allowed for under its code.

Under the IMC code, a company could be fined a maximum of $250 000 for four violations in a single year. In 2016, IMC companies spent between $2.121 to $13.994 million on each of the top 50 most promoted drugs.^14^ A fine of $250 000 would only add between 11.8 percent (250 000/2 121 000) to 1.8 percent (250 000/13 994 000) to the cost of promotion for that product, an amount that might be insufficient to deter companies from violating the IMC code.

The PAAB did require the immediate withdrawal of material several times, but it does not release any information about how long the material had been in use before it was withdrawn. Marketing campaigns have a shelf-life, and the promotion may already have had its desired effect by the time it was withdrawn. Therefore, there would not be any adverse consequences from the imposition of the penalty. Issues that PAAB cannot resolve or that fall outside of its mandate are forwarded on to Health Canada for resolution, but PAAB does not report on Health Canada's actions and therefore the value of escalating complaints is not known.

### Compliance

The IMC code requires company employees, including sales representatives, to “provide full and factual information on products, without misrepresentation or exaggeration. Statements must be accurate and complete. They should not be misleading, either directly or by implication”.^11^ A study based on primary care physicians in Vancouver and Montreal filling out questionnaires after a visit from sales representatives found that no safety information was provided in almost two-thirds of visits; serious adverse events and contraindications were only mentioned 5 percent and 14 percent of the time, respectively; and sales representatives mentioned unapproved indications in 13 percent of their visits.^37^ Sales representatives in these two cities also exaggerated the benefits and played down the harms of opioids.^38^ Similar findings about the provision of safety information were reported in Australia, where the provision of information on product risk was minimal despite the Code of Conduct from the Australian Pharmaceutical Manufacturers Association (now Medicines Australia) stating that promotional material “must be current, accurate and balanced, must not mislead, either directly, by implication, or omission”.^39^ Self-regulatory codes from other high-income countries have failed to ensure that claims in ads are supported by high quality evidence.^40–42^

### Complaints and Codes

The low number of recorded complaints and the even lower number of upheld complaints are likely due to a combination of weaknesses in the two Canadian codes: closed administration, poor monitoring, and weak penalties, the latter contributing to poor compliance. The value of self-regulation has also been questioned in other countries. After an examination of the pharmaceutical industry, the Health Committee of the U.K. parliament concluded that “the examples cited to us of breaches of advertising regulations, cover-up of negative medicines information and provision of misleading information to prescribers suggest that self-regulation [by the UK pharmaceutical industry] is not working satisfactorily”.^43^ The inadequacy of self-regulation was reinforced a decade later by a study of data from self-regulatory bodies in Sweden and the United Kingdom on complaints, complainants, and rulings for the period 2004–2012. The agencies in these two countries have been cited as good examples of self-regulation by industry^44,45^ and regulators.^4,46^ The authors interpreted their evidence as showing that “self-regulation has failed to sufficiently deter industry from engaging in frequent and sometimes serious unethical practices, as judged by the industry's own standards”.^19^

Another factor that needs to be considered in explaining the low number of recorded complaints is the absence of any complaints coming from internal company sources. Canada lacks the equivalent of the American *Qui Tam* legislation that provides an incentive for whistleblowers to come forward by rewarding them with a portion of the monies recovered through successful legal action taken up the federal government in that country.^47^

### Limitations

The results of this study do not apply to the regulation of direct-to-consumer advertising, promotion of over-the-counter medicines, or jurisdictions where promotion is directly regulated by government. The number of actual violations of the IMC and PAAB codes is likely to be an underestimate because both organizations use a passive system of monitoring. This study accepted the rulings about whether the codes had been violated. Finally, all the information was collected by a single person and that may have introduced biases, although information about complaints and violations was only transcribed.

## Conclusion

Putting the low number of complaints in the context of weak codes, closed administration, poor monitoring, inadequate penalties, and poor compliance, the IMC and PAAB codes are failing to ensure that Canadian physicians are receiving the accurate and complete evidence that is needed to help ensure appropriate prescribing. There are several interim measures that can be taken to improve the situation, including strengthening the codes; involving outside organizations that are free of conflict-of-interest in governing and administering the codes; and adopting active monitoring to look for violations and imposing meaningful penalties–for example, not being able to promote the product for a period of time. However, despite the evidence showing that voluntary and industry codes are ineffective, these measures or comparable ones have not been adopted in any country. Moreover, even if they were adopted, it is likely that companies would be able to find ways around them. The longer-term solution is to entirely remove regulation of promotion from industry and rest it in an independent body that is established through legislation and has ongoing stable funding to perform its task. In addition to independent regulation, two other measures should be instituted. First, even the best external regulation can be ineffective in controlling internal company practices that ultimately result in deceptive promotion. The counter to this problem is measures to encourage whistleblowing. Second, physicians must also be provided with free or low-cost sources of independent objective information that will reduce the temptation to turn to industry for information. A combination of independent regulation and independent information should substantially improve prescribing.

## Supplemental Material

sj-pdf-1-joh-10.1177_27551938231165158 - Supplemental material for Complaints about Violations of Voluntary and Pharmaceutical Industry-Run Medicine Promotion Codes in Canada Click here for additional data file. Supplemental material, sj-pdf-1-joh-10.1177_27551938231165158 for Complaints about Violations of Voluntary and Pharmaceutical Industry-Run Medicine Promotion Codes in Canada by Joel Lexchin in International Journal of Social Determinants of Health and Health Services

sj-pdf-2-joh-10.1177_27551938231165158 - Supplemental material for Complaints about Violations of Voluntary and Pharmaceutical Industry-Run Medicine Promotion Codes in Canada Click here for additional data file. Supplemental material, sj-pdf-2-joh-10.1177_27551938231165158 for Complaints about Violations of Voluntary and Pharmaceutical Industry-Run Medicine Promotion Codes in Canada by Joel Lexchin in International Journal of Social Determinants of Health and Health Services
