# Supplementary material for: Complaints about Violations of Voluntary and Pharmaceutical Industry-Run Medicine Promotion Codes in Canada
Source: Int J Soc Determinants Health Health Serv. 2023 Mar 20;53(4):518–27. doi: 10.1177/27551938231165158 (PMC10631264; doi:10.1177/27551938231165158)
Supplement: sj-pdf-1-joh-10.1177_27551938231165158 - Supplemental material for Complaints about Violations of Voluntary and Pharmaceutical Industry-Run Medicine Promotion Codes in Canada [file sj-pdf-1-joh-10.1177_27551938231165158.pdf]

Supplementary File 1: Complaints about violations of the Innovative Medicines Canada Code of Ethical Practices

Year	Parties	Issue	Decision
2012	Individual X, patient group members vs. Company Y	Engaged in disguised marketing to patient group members in contravention of Section 12	No breach
2013	No complaints		
2014	No complaints		
2015	Company X vs. Company Y	Company Y nduced group purchasing by providing inappropriate incentives and violates guiding principle 8 and section 12.2.4	No breach
2016	Company X vs. Company Y	Company Y's patient support program violates Guiding Principles 2, 3, 8 and sections 2.1, 14.1.1, 14.2.2. and 14.2.3	No breach
	Company C vs. Compar	Company D's patient support program violates Guiding Principles 2, 3, 8 and sections 2.1, 14.1.1, 14.2.2. and 14.2.3	No breach
	Company A vs. Company B	Company B's patient support program violates Guiding Principles 2, 3, 8 and sections 2.1, 14.1.1, 14.2.2. and 14.2.3	No breach
2017	No complaints		
2018	Company X vs. Company Y	Company Y's patient support program violates Guiding Principles 1.1 and sections 2.1, 14.1.1, 14.2.2. and 14.2.3	No breach
2019	Nothing recorded		
2020	Nothing recorded		
2021	Nothing recorded		
