# Supplementary material for: Complaints about Violations of Voluntary and Pharmaceutical Industry-Run Medicine Promotion Codes in Canada
Source: Int J Soc Determinants Health Health Serv. 2023 Mar 20;53(4):518–27. doi: 10.1177/27551938231165158 (PMC10631264; doi:10.1177/27551938231165158)
Supplement: sj-pdf-2-joh-10.1177_27551938231165158 - Supplemental material for Complaints about Violations of Voluntary and Pharmaceutical Industry-Run Medicine Promotion Codes in Canada [file sj-pdf-2-joh-10.1177_27551938231165158.pdf]

Supplementary File 2: Complaints about violations of the Pharmaceutical Advertising Advisory Board Code of Advertising Acceptance

Year	Parties (Complainant, Company subject to complaint)	Preclearance	Allegation	Issue	Decision	Penalty & appeal
2012	Sanofi vs Pfizer	Yes	Misleading statements	Statements about Pradax and patient safety allegations in textbook "Managing Pain"	Upheld	Cease distribution immediately and replace material as necessary. No appeal.
	Private physician vs Neilmed	No	12-02 Neilmed Nasal Spray samples	Samples distributed illegally	Sent to Health Canada for investigation	Not documented
	Two private physicians vs Purdue	No	Textbook “Managing Pai	Misleading statements and patient safety allegations in textbook "Managing Pain"	Sent to Health Canada for investigation	Not documented
	Bayer vs Boehringer	Yes	Pradax journal advertisement	Claim that Pradax reduces risk of intracranial bleeding by 59% is not an indication	Upheld	Withdraw journal ad and any other relevant APS. Send PAAB letter including action plan and timeline. No appeal.
		Yes		Ad focuses on 150 mg dose and doesn't comment on 110 mg dose	Upheld	Withdraw journal ad and any other relevant APS. Send PAAB letter including action plan and timeline. No appeal.
2013	Experichem vs Oral Dent	No	Periplus brochure	Misleading claims	Product not approved for use in Canada and complaint referred to Health Canada	Not documented
	Grifols vs Octapharma	No	Multiple APS	misleading claims, no indication statement, comparative, DTC violations	Upheld	Send the alleged DTC violations to Health Canada. Multiple violations required shutdown of web-site and cease distribution of letters and posters. Submit for PAAB review. No appeal.
2014	Boehringer vs. Bayer	Yes	Xarelto detail aid	Visual layout of dosing recommendations is misleading	Dismissed	$500 registration fee assessed to Boehringer. No appeal.
				Recommended dose based on patient's age not presented accurately	Dismissed	$500 registration fee assessed to Boehringer. No appeal.
				Dosing selection algorithms complicates process and intentionally misleads physician	Dismissed	$500 registration fee assessed to Boehringer. No appeal.
	No information on one complaint					

2015	Sanofi vs Pfizer	Yes	Epipen APS various	The Pfizer Canada 2 Steps Campaign provides misleading and incomplete information in violation of the PAAB Code	Dismissed	$500 registration fee assessed to Sanofi. No appeal.
	Pfizer vs GSK	Yes	Votrient APS	APS contains misleading claims that are based on inadequate supporting material and statistics that are not presented in a clear and transparent manner	Upheld	GSK will be required to add additional footnotes in future APS to help improve the context of the claimNo appeal.
	BioSyent vs Odan	No	promotional systems for Odan Polysaccharide Iron Complex	Odan claimed its product was comparable from a therapeutic perspective to BioSyent's	Upheld	No appeal.
	Biosyent vs Medical Futures	No	3 promotional detail aids distributed to pharmacist	misleading claims regarding “optimal iron” and alleged potentially misleading comparative claims vs Feramax	Upheld.	MFI to cease and desist the alleged claims and recall material if distributed to health professionals. No appeal.
	Serono vs Biogen	No	Biogen APS	Multiple code violations	Upheld	Biogen should cease distributing the promotional materials in question and materials with similar content alleged to be in violation of the PAAB Code of Advertising Acceptance. Biogen should recall and retrieve such material from the marketplace. We invite Biogen to preclear all APS through the PAAB. Biogen should inform me in writing no later than Wednesday July 22, 2015 that they will comply with this ruling and give an action plan with dates of how they will remove the violative material from the marketplace.If Biogen does not agree to the above request, I will transfer this complaint to Health Canada and request enfo[illegible]
	Biogen vs Novartis	No	Internal training document that was used as product promotion by a sales representative	Novartis intentionally distributed internal document.to a physician	Upheld	None documented. No appeal.
				Novartis document contains inaccuracies disparaging Biogen's product	Upheld	None documented. No appeal.

2016	Biosyent vs Ethical Remedies	No	EBMFer (NPN80042242) APS	Superiority claims for product	Upheld	APS is in violation of PAAB Code and should not be used. PAAB wants evidence that material removed from sales reps. No appeal.
	Novo Nordisk vs Searchlight Pharma	No	Estragyn leave behind	Price chart constitutes off-label promotion and is misleading.	Upheld	Proof of actions taken by Searchlight Pharma with respect to controlling activities of sales reps. No appeal.
				Price chart uses outdated and misquoted statements from the North American Menopause Society	Upheld	Proof of actions taken by Searchlight Pharma with respect to controlling activities of sales reps. No appeal.
				Price chart fails to disclose that an appropriate dose of progestin is required	Upheld	Proof of actions taken by Searchlight Pharma with respect to controlling activities of sales reps. No appeal.
	Tribute Pharmaceuticals vs Vanc Pharmaceuticals	No	Hema-Fer promotional materials	Type and quantity of iron in a single tablet of Hema-Fer.	Upheld	If PAAB did review promotional materials it would ask for "cover to cover" revisions. PAAB requesting Health Canada to expedite review of complaint and bring appropriate enforcement. No appeal
	Septodont vs Pierrel	No	Orabloc ad in Oral Health	No fair balance, superlative claims unsubstantiated	Upheld	Health Canada informed. No appeal
2017	Amgen vs Merck	No	Interview article in "Biotechnology Focus" promoting Brenzys	Interview does not fall under any of the prescribed categories of exemptions from the Code and violates multiple sections of the Code.	Health Canada consulted and confirmed that it considered the item to be advertising. PAAB agreed that item was advertising.	APS should have been submitted for review. Merck filed Stage Three Appeal. Panel upheld commissioner's ruling
	Roche + Janssen + AbbVie + Takeda vs Merck	No	Editorial Advertising “The Rise of Biosimilars” appearing in “Benefits Canada” December 2016	Report falls within scope of Code and none of the exemptions apply. Multiple sections violated.	Upheld. DTC portion referred to Health Canada	The PAAB has ruled that the item is an advertising/promotional system subject to review and there are alleged violations of safety and efficacy statement provisions in the PAAB code. Website corrected by Merck. Merck filed and then withdrew Stage Three Appeal
	Lundbeck vs Allergan	No	Fetzima Detail Aid	Allergan has not proven an exclusive "only" claim.	Upheld	File was referred to Health Canada because of non-compliance with previous PAAB rulings. Pending Health Canada investigation and decision.

			Claim based on a review article	Upheld	File was referred to Health Canada because of non-compliance with previous PAAB rulings. Pending Health Canada investigation and decision.
			Statement links non-clinical parameters to clinical benefits.	Upheld	File was referred to Health Canada because of non-compliance with previous PAAB rulings. Pending Health Canada investigation and decision.
			Claim is too broad and not reflective of results of one study	Upheld	File was referred to Health Canada because of non-compliance with previous PAAB rulings. Pending Health Canada investigation and decision.
			Misleading bar graph	Upheld	File was referred to Health Canada because of non-compliance with previous PAAB rulings. Pending Health Canada investigation and decision.
			Wording is not completely reflective of the wording in the product monograph	Upheld	File was referred to Health Canada because of non-compliance with previous PAAB rulings. Pending Health Canada investigation and decision.
			Representative contact information added	PAAB has no objection as addition does not alter context of APS	File was referred to Health Canada because of non-compliance with previous PAAB rulings. Pending Health Canada investigation and decision.
			Claim not supported by Product Monograph	Upheld	File was referred to Health Canada because of non-compliance with previous PAAB rulings. Pending Health Canada investigation and decision.
			Timing issues of first marketing	Unclear	File was referred to Health Canada because of non-compliance with previous PAAB rulings. Pending Health Canada investigation and decision.
Novartis vs Bayer	No	Now I Know website and Leaflet in support of Eyelea	PAAB should have reviewed website and multiple misleading claims alleged	Upheld	Leaflet should be submitted for review. Webiste is DTCI and does not fit into Code.Bayer should submit similar material to the PAAB in the future. No appeal

	Aralez vs Pediapharm	No	Rupall detail aid	Preclearance review service was not used by Pediapharm and that is a violation of the PAAB code.	Upheld	The APS in question is clearly in violation of the PAAB Code and would have benefited from a PAAB preclearance review. I request Pediapharm to withdraw this APS from the marketplace and stop further distribution. I would like to see an action plan and messages directed at Pediapharm staff to withdraw this APS and agreement to stop further distribution. No appeal.
2018	Pediapharm vs Tribute	Yes	Blexten Leave Behind and Booth Panel	Content of the Blexten promotional material is vague and does not reflect all the cautionary information from the Blexten product monograph	Upheld.	PAAB will not renew the current APS when they expire. We encourage Tribute to address allegation #1 as soon as is possible to avoid the perception of misleading advertising. I would like to hear a response from Tribute within 5 business days of electronic receipt of this ruling. If I receive no response as they indicated in their stage one behaviour, I will forward the complaint to Health Canada
				Claim not reflect the limitations of the product monograph	Rejected	
				Claim can mislead healthcare professionals	Rejected	
				Pharmacodynamic section ignores the negative findings that should be included	Rejected	
	Aralez vs Pediapharm	Yes	Pediapharm Rupall leave behind	The dosage information in this APS intentionally omits important information that is found in the Dosing Considerations of the Product Monograph	Rejected	No reason to change the ruling of the reviewer's decision regarding the fair balance issue raised by Aralez. PAAB will issue an invoice to Aralez in the amount of $500 for the registration fee of this complaint.

	Biosyent vs Volo Healthcare	No	Optifer Alpha Detail Aic	Misleading claims	Upheld	1. Stop distribution of said material to the representatives;<br>2. Mandate to representatives to stop using said material immediately;<br>3. To collect and destroy said material that they find in any HCP offices. No appeal
2019	AbbVie vs. Gilead	Yes	Epclusa	Side by side presentation of number of pills and number of product packages in a treatment course misleading	2 of 9 allegations upheld	The advertiser was requested to cease use of the APS and resubmit a revised version of the APS for approval. No appeal
	Amgen vs. Pfizer	No	Inflectra	Email sent to healthcare professionals alleged to be advertising and broaden indications	2 of 2 allegations upheld	The advertiser had already clarified that the email is no longer in use. Therefore, no immediate action was requested. However, the advertiser was informed that similar future emails should be submitted for preclearance. No appeal
	Sanofi vs. Sequiris	No	Fluad	APS was advertising	Upheld 7 of 7 allegations	Advertiser was requested to cease distribution of the APS and submit a revised version of the APS for preclearance. No appeal
	AKCAE vs. Alnylam	No	Onpattro	APS claim in conference booth that advertiser's product changes "the natural course" of the indicated condition was deemed to be insumciently substantiated.	Upheld 5 of 8 allegations	The advertiser was requested to cease use of the APS. The PAAB strongly recommended that Alnylam begins submitting advertisng to PAAB for preclearance. No appeal.
	Medexus vs. Aralez	Yes	Blexten	Not clear	Rejected	No appeal.
	Physician vs Bayer	Yes	Aspirin	No use of aboslute terms	Upheld	Referred to Health Canada which will request Bayer to make changes to Product Monograph. Aadvertiser was requested to resubmit a revised version of the APS for approval. No appeal.

	Replete vs. BioSyent	No	RFeraMax	Accredited CME videos were placed on a promotional website and therefore subject to advertising regulations. APS contained unsubstantiated claims	Upheld 11 of 14 allegations	Advertiser was requested to immediately take down the HCP section of the website and to perform the necessary revisions. No appeal.
	Lupin vs. Allergan	No	Constella & Viberzi copromotional APS	Callouts were associated with this APS presentation in a manner which could cause the reader to incorrectly presume that those callouts originated from the guideline's algorithm. Even if callouts were lifted from guideline's algorithm they would not have been accepted in advertising	Upheld 3 of 4 allegations	The advertiser was requested cease use of any affected APS and to submit revised versions to PAAB for review.
	BioSyent vs. Replete	No	Ferapro	APS misleading	Upheld 5 of 7 allegations	The advertiser was requested to cease use of the APS and submit a revised version of the APS for approval. No appeal.
2020	Healthcare professional vs Pfizer	Yes	Relpax and Xalatan/Xalacom	Messaging in ads suggests that the patient is assured to receive the brand-name product if the prescriber writes "no sub".	Upheld 2 of 2 allegations	A two pronged approach will be applied to improve future versions of APS with similar "no sub" messages across the the manufacturer's product portfolio (and across the the entire industry) so as not to suggest that the patient is assured to receive the brand-name product where this is not necessarily the case. No appeal
	Gilead vs. AbbVie	No	Maviret	Wi-Fi materials were not exempt from preclearance	Upheld 2 of 2 allegations	Given the advertiser's history of preclearance and adherence to the PAAB code, AND given that the Wi-Fi materials were no longer in use, it was deemed that there was no need to impose additional PAAB sanctions. No appeal

2021	Healthcare professional vs Otsuka & Lundbeck	Yes	Rexulti	Advertisement did not include a reference list	Upheld 1 of 3 allegations	No immediate action was required. However, the ruling indicated that the PAAB will require insertion of the reference citation upon resubmission of the advertisement for preclearance. No statement about whether ruling appealed.
	Leo Pharma vs. Bausch Health	No	Duobril	Communication materials that were part of a virtual presentation that was organized by the advertiser were advertising	Upheld 2 of 2 allegations	The advertiser was to confirm that the invitation and slides would not be used in the future. Additionally, in the spirit of promoting self-regulation, the Commissioner was to be provided an outline of the steps to be undertaken to avoid recurrence of similar instances in the future. No statement about whether ruling appealed.
	Healthcare professional vs Eisai	No	Dayvigo	Problems with journal ad that was not approved by PAAB	Upheld 3 of 4 allegations	To pull the ad immediately. Distribution did not resume until the ad was precleared by PAAB. No statement about whether ruling appealed.
	Amgen vs. Viatris	No	Hulio	Misleading claims	Upheld 2 of 2 allegations	Pull the ad immediately. No statement about whether ruling appealed
	Miravo vs. Medexus	Yes, but expi	Rupall detail aid	Poetentially misleading claim	Partly upheld 5 allegations	Cease distribution of the ad (distribution may have already ceased given the expired approval period). PAAB notified the advertiser that the corrections would need to be made through the preclearance process prior to resumption of distribution/dissemination of the ad. No statement about whether ruling appealed
